# Coping with family structure in genome-wide association studies: a comparative evaluation

**DOI:** 10.1186/s12919-018-0151-8

**Published:** 2018-09-17

**Authors:** Yayun Hsu, Jonathan Auerbach, Tian Zheng, Shaw-hwa Lo

**Affiliations:** Department of Statistics, Columbia University, 1255 Amsterdam Avenue, New York, NY 10027 USA

## Abstract

In this paper, a fully statistical investigation of the control of family structure as random effects is analyzed and discussed, using both the genome-wide association studies (GWAS) data and simulated data. Three modeling strategies are proposed and the analysis results suggest the hybrid use of results from all possible models should be combined in practice.

## Background

It is well known that genome-wide association studies (GWAS) may lead to spurious findings if one fails to address the dependence among individuals such as that resulting from family structure. If the true dependence structure is known, the best practice is to explicitly incorporate this information into the analysis. However, the true dependence structure is rarely available. Consequently, different strategies have been proposed to address this issue [1–13].

Statistically, we use multilevel models to model complicated family structures. Multilevel models, also known as variance component models, random effects models, or hierarchical linear models, have seen rapid growth and development in many different fields [14]. Multilevel models provide a flexible regression modeling framework for handling data sampled from clustered population structures, such as students within classes that are within schools, patients within hospitals, repeated measurements within individuals, or children within families. Ignoring the multilevel structure of the data can lead to incorrect inferences that result from underestimating the standard errors for the regression coefficients.

Linear mixed-effect regression models assume the family effect to be a random effect. The covariance structure for the random effect is generally assumed to correspond to that implied by a polygenic model, incorporating the genetic relationship (kinship) between each pair of individuals. Although the use of this linear mixed-effect regression model was originally proposed for pedigrees with known relationships [1–5], this approach is popular for use with samples of unknown or uncertain relationship [6–13], including apparently unrelated samples that may nevertheless display distant levels of common ancestry.

The data set we use for this analysis is collected under the Genetics of Lipid Lowering Drugs and Diet Network (GOLDN) study. It was designed to identify genetic determinants of lipid response to two interventions: (1) a high-fat meal challenge and (2) fenofibrate treatment for 3 weeks [15]. The dataset only includes families with at least two siblings. This family information allow us for the analysis of family structural dependencies. Volunteers were required to withhold lipid-lowering agents (pharmaceuticals or nutraceuticals) for at least 4 weeks prior to their initial visit. A total of 1053 met all eligibility requirements. For the current study, we evaluated fasting triglyceride (TG) and very-low-density lipoprotein cholesterol among 991 participants for whom epigenetic data were available [16].

## Methods

### Model setup and statistical analysis

Using a linear mixed-effect model, we model the response to both the fixed effects *X* and random effects *Z* as follows:

1 $$ y= X\beta + Z\mu +\epsilon $$

2 $$ \mu \sim N\left(0,{\sigma}_{\mu}^2{I}_m\right) $$

3 $$ \epsilon \sim N\left(0,{\sigma}_{\varepsilon}^2{I}_n\right) $$

where *y* is the vector of observations, with mean *Xβ*; *β* is an unknown vector of fixed effects; *μ* is the vector of unknown random effects, with mean 0 and the variance–covariance matrix proportional to the kinship matrix; and *ϵ* is an unknown vector of random errors, with mean 0 and the variance–covariance matrix assumed to be proportional to the identity matrix. *X* and *Z* are design matrices and *I*_*m*_ and *I*_*n*_ are identity matrices.

The variance–covariance matrix of *y* is given by

4 $$ Cov(y)={\sigma}_{\mu}^2\ Z{Z}^{\prime }+{\sigma}_{\varepsilon}^2{I}_n $$

Analysis of variance is one of the popular methods in the statistical literature to estimate the variance components ${\sigma}_{\mu}^2$ and ${\sigma}_{\varepsilon}^2$.

**Fig. 1:**
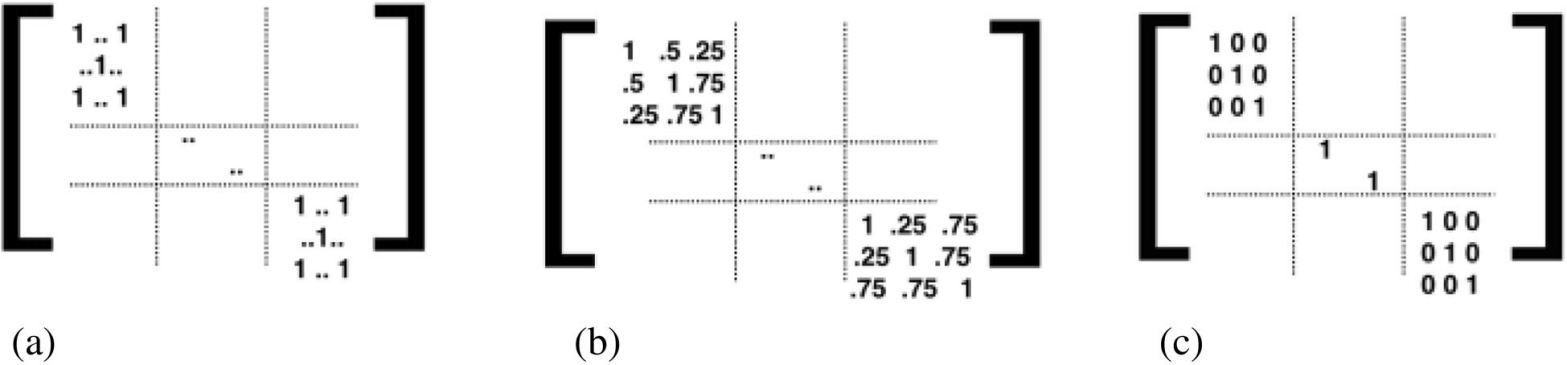
Kinship coefficient matrices used in 3 modeling options for fitting the linear mixed-effects model to data with family structures. **a** Option 1: representatives. **b** Option 2: kinship. **c** Option 3: independent

In a mixed-effect model, one needs to consider a covariance matrix for the random effects. In this study, family structures provide additional information on potential dependence among individuals. We attempt three different strategies to account for family structures. First, we recreate the Irvin study [13]: modeling the beta score as a function of TG level using mixed linear regression adjusted for age, gender, study site, and 4 methylation principal components as fixed effects [namely, the *X* in eq. (1)] and the family structure [the *Z* in eq. (1)] as random effects. Throughout the 3 models we try, the matrices *X* are identical, as in [13]. We made this decision because our interest is in the evaluation of the covariance matrix introduced by the random effects *Z*, but not the fixed part *X*. Here, the kinship matrix is based on the theoretical estimates—the probability of sharing genetic relatedness. We refer to this *ideal* option as the *kinship* option. When the kinship information is not available, there are two other coping strategies: one that assumes everyone in this study is independent, ignoring family structure (denoted as the *independence* option), and one that uses only one randomly drawn representative individual from each family (referred as the *representative* option). While these 3 modeling options differ in the covariance matrix for the random effects, the fixed-effect parts remain the same. Figure 1 illustrates the assumed variance–covariance matrices.

Option 1, in expectation, is equivalent to using centroids for each family, which is not always feasible for all data types.

**Fig. 2:**
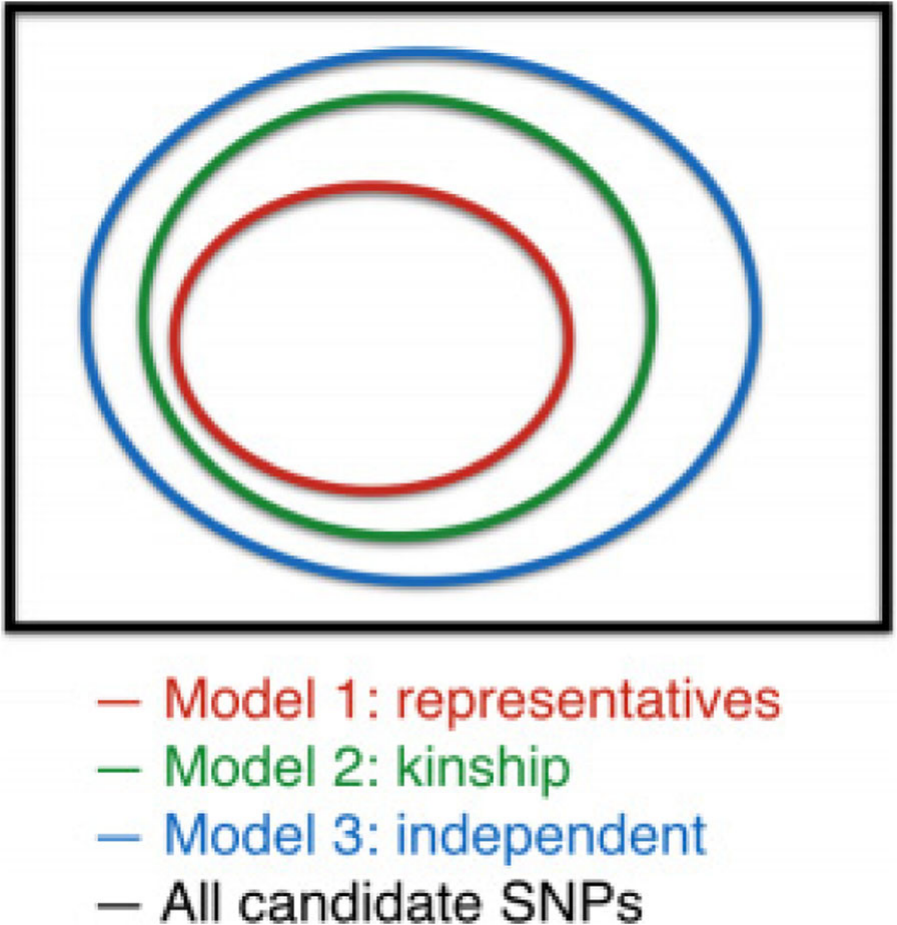
Ideal relation among significant SNP sets from 3 different modeling options

Under the assumption that the assumed model is correct, the important single-nucleotide polymorphisms (SNPs) identified by these 3 modeling options should have a nested structure as in Fig. 2 in expectation, where the black box includes all SNPs and each circle includes the significant SNP sets concluded from each model. In practice, we also expect that the random variations in data and departures from model assumptions will lead to fewer overlapped identified SNP sets. If the analysis results from real data meet these expectations, it is only a matter of tradeoff between effective sample size and power. If any results from real data analysis deviates from this theoretical expectation, it might suggest the need for more detailed inspection of the coping strategies for family structures.

We use the *lmekin* function in R package *coxme* for the estimation of the mixed effect model and compare the results from the 3 modeling options. This is done by using user-specified variance functions. We use the 3 random effect structures explained previously in Fig. 1 for the simulation. The results from the application to the GAW data set and a simple simulation study are shown and discussed in the next section.

## Results

### Application to GAW20 data set

**Fig. 3:**
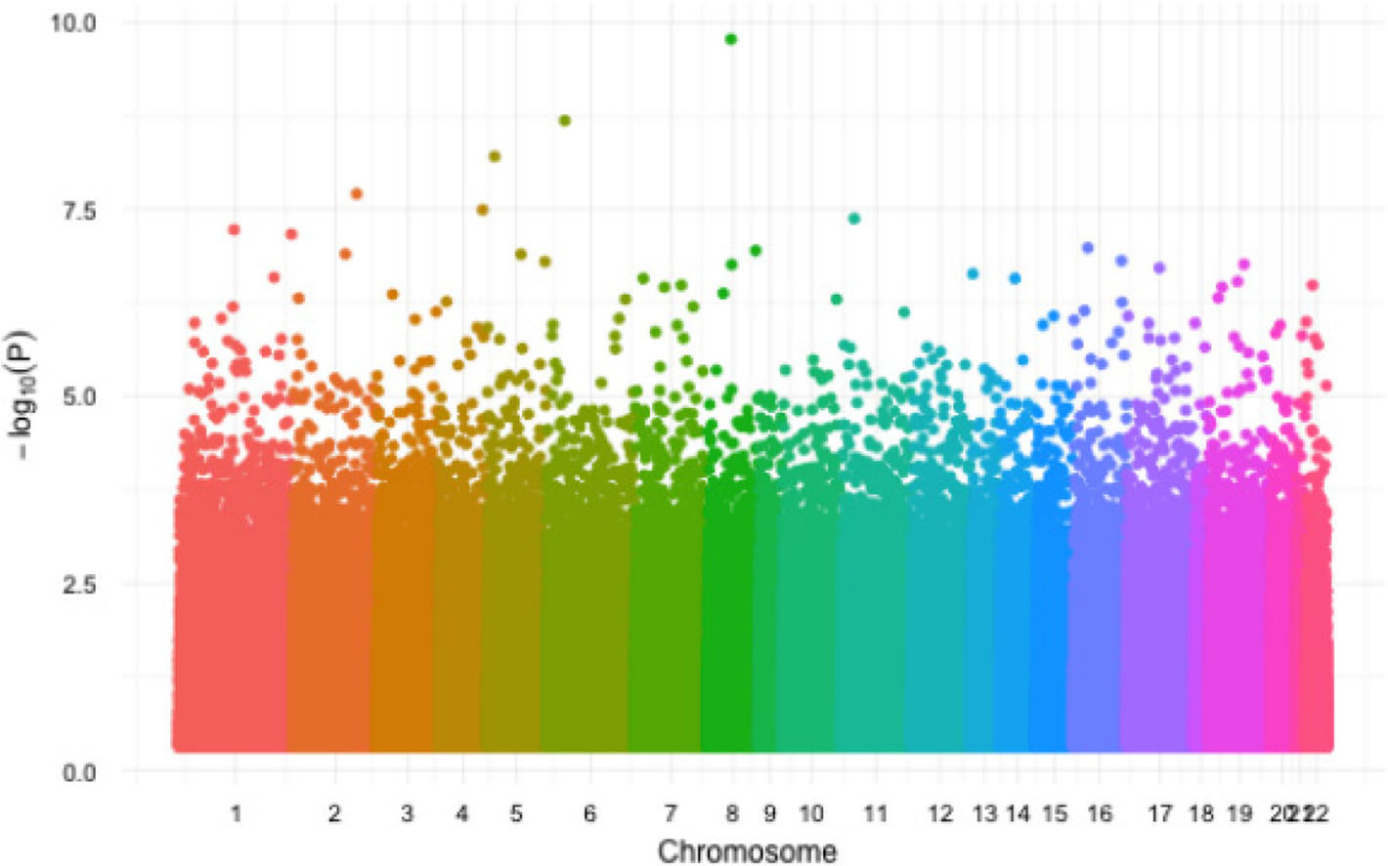
Manhattan plots for option 1

**Fig. 4:**
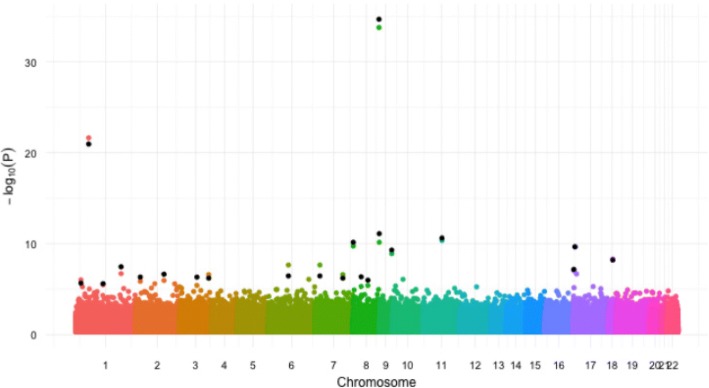
Manhattan plots for option 2 and option 3 (*black dots*)

We first apply the 3 modeling options for fitting linear mixed effects model to the GAW20 data. For each SNP, as in Irvin et al. [13], we test for statistical significant departure from the null hypothesis that the SNP has no effect on the beta scores. The Manhattan plots from these 3 modeling options are shown in Figs. 3 and 4. As expected, when the effective sample size is small, as with the representative option, we have less power in detecting significant SNPs. So the overall *p* values from option 1 are lower than those from options 2 and 3. Figure 4 plots the results from options 2 and 3 together, because of their similarity. While the chromatic dots show the results using the original Irvin et al. study [13], the black dots are the significant findings from option 3. The results from these 2 options are very similar. In fact, the top 100 significant SNPs identified using options 2 and 3 have 71 SNPs in common. Using option 1, cg12033043 from chromosome 8 is found to be the most significant SNP. Using option 2, cg27026926 from chromosome 8, cg05599320 from chromosome 1, cg13982695 from chromosome 11, cg27331738 from chromosome 8, and cg00223867 from chromosome 8 are identified to be the top 5 significant SNPs, which are among the top 100 SNPs identified by option 3.

**Fig. 5:**
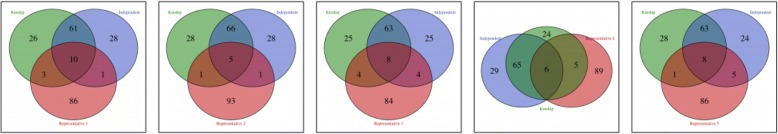
Significant SNP sets identified from 3 different models (top 100) from 5 random runs. For each run, 1 individual was randomly drawn from each family, which led to different results. The results from the kinship and the independence options remain unchanged. (Coloring: red for *representative*, green for *kinship*, and blue for *independence*)

To average out random variation resulting from change, we randomly choose 5 sets of representatives (one from each family for each set). Figure 5 shows Venn diagrams of the overlaps in findings from the 3 modeling options. Note that there is only one set of results from the independence option and the kinship option. Five different sets are derived from 5 randomly drawn sets of individuals for the representative option, which lead to a different level of overlap with the other two options. The numbers represent the number of overlapping SNPs.

For each run, one individual was randomly drawn from each family, which led to different results. The results from the kinship and the independence options remain unchanged. (Coloring: red for *representative*, green for *kinship*, and blue for *independence*.)

This result does not completely meet our expectation. Both option 1 and option 2 are fitting statistical valid models to the data, while option 3 (independence) is based on an incorrect assumption. We observe from Fig. 5 that results from option 1 are highly variable and overlap very little with those from option 2. Conversely, even though option 3 is under an incorrect assumption, it has a substantial overlap with option 2.

### Simulation studies

**Fig. 6:**
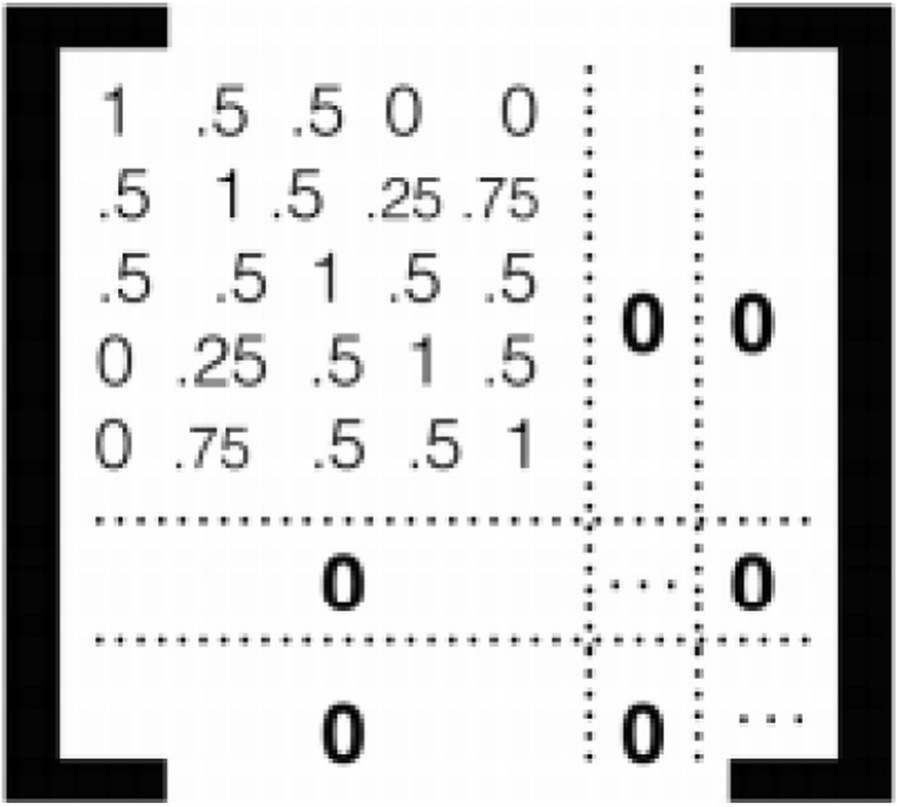
Kinship coefficient matrix used in the simulation study for family structures

**Fig. 7:**
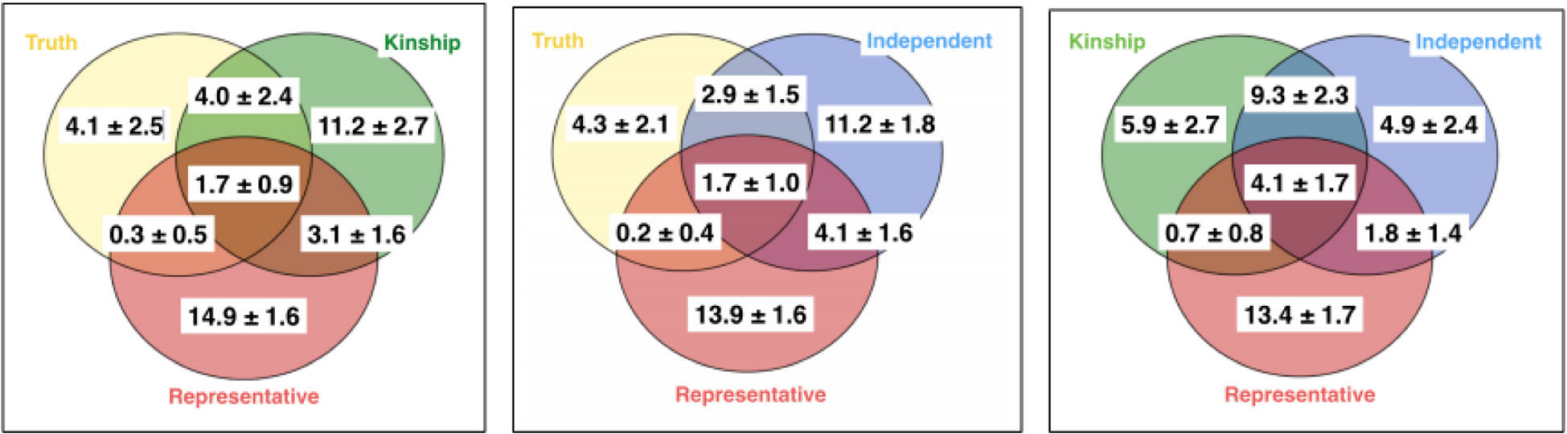
Simulation results: Significant SNP sets identified from 3 different models (top 20)

To understand what we observed in the real data analysis, we carry out a simple simulation study to further compare the three coping options. We ran independently 3 sets of simulations, in which we draw samples under the model assumed in eq. (1) to eq. (3), with random effects from multivariate normal distribution and known variance–covariance structure. Among the 1000 SNPs simulated, only 10 are under the alternative hypothesis that the mean of the responses is not zero. We simulated 1000 individual SNPs within 200 families. Figure 6 shows the kinship coefficient matrix used in the simulation. Venn diagrams in Fig. 7 summarize the results from these 3 simulations; the numbers show the “average counts ± SD.” First, we can see that option 2 (the *kinship* option) provides the best performance, with the largest overlap with the true signals. Second, in the third Venn diagram we obtained, we see the same phenomenon as in the real data analysis; that is, the findings from the representative option is further away from the independent option to the kinship option. Conversely, the overlapped results from the representative option and the independence option have, on average, slightly lower false discovery rates. This suggests a potential hybrid modeling option that combines results from the independence option and the representative option when the kinship coefficients are not available.

## Discussion and conclusions

In this paper, we evaluate different modeling options for coping with data with family structure in the context of genetics studies. Our analysis suggests the need for adjusting for kinships. When kinship information is not known, we compare two opposite strategies, one that treats all individuals in the study as independent and the other that approximately uses the family centroid by randomly sampling 1 representative from each family. Our results suggest that the cost of ignoring other members from a family (the *representative* option) is greater than that of ignoring dependence among all individuals in a study (the *independence* option). More research should be conducted to understand this phenomenon. From the results of a simple simulation, we suggest that both strategies should be used in practice and that the focus should be on SNPs that are identified by both.
